# Nano zinc elicited biochemical characterization, nutritional assessment, antioxidant enzymes and fatty acid profiling of rapeseed

**DOI:** 10.1371/journal.pone.0241568

**Published:** 2020-11-10

**Authors:** Sohail, Khalid Kamran, Birgit Kemmerling, Meshal Shutaywi, Zia ur Rehman Mashwani

**Affiliations:** 1 Department of Botany, PMAS Arid Agriculture University, Rawalpindi, Pakistan; 2 ZMBP–Department of Plant Biochemistry, University of Tuebingen, Tuebingen, Germany; 3 Department of Biological Sciences, University of Lakki Marwat, Lakki Marwat, Pakistan; 4 Department of Mathematics, College of Science & Arts, King Abdulaziz University, Rabigh, Saudi Arabia; Government College University Faisalabad, PAKISTAN

## Abstract

The use of nanomaterials in agriculture is a current need and could be helpful in overcoming food security risks. *Brassica napus* L. is the third most important crop for edible oil, having double low unsaturated fatty acids. In the present study, we investigated the effects of green synthesized Zn NPs on biochemical effects, antioxidant enzymes, nutritional quality parameters and on the fatty acid profile of rapeseed (*B*. *napus*). Plant-mediated synthesis of zinc nanoparticles (Zn NPs) was carried out using *Mentha arvensis* L. leaf extract followed by characterization through ultraviolet–visible spectroscopy (UV-vis), scanning electron microscopy (SEM), transmission electron microscopy (TEM), energy dispersive X-Ray (EDX), and X-Ray diffraction (XRD). NPs exhibited irregular shapes ranging in size from 30–70 nm and EDX analysis confirmed 96.08% of Zn in the sample. The investigated biochemical characterization (protein content, proline content, total soluble sugar (TSS), total flavonoid content (TFC), and total phenolic content (TPC) showed a substantial change on exposure to Zn NPs. A dose-dependent gradual increase was observed in the antioxidant enzymes, superoxide dismutase (SOD), peroxidase (POD), and catalase (CAT). Oil and moisture contents dropped significantly from the control level in the rapeseed (*B*. *napus*) varieties. However, different trends in nutritional (Zn, Na+, K+) and fatty acid profiling of *B*. *napus* have been noted. This study demonstrates that Zn NPs have the potential to improve the biochemical, nutritional, antioxidant enzymes, and fatty acid profile of *B*. *napus* varieties.

## Introduction

With the progression of nanotechnology, the number of nanoparticles (NPs) used in consuming products has increased dramatically [1]. Nanotechnology is regarded as a 21st-century science, which has applications in many fields, i.e., biology, chemistry, and agriculture [1, 2]. Metal NPs are widely used in commercial applications ranging from coating, topical sunscreens, soaps, antimicrobial agents and in agricultural applications [3]. With the large production of metallic NPs and their use in daily usable products, environmental contamination is becoming an important matter of concern [4].

Nanotechnology has enormous potential to modify conventional agricultural practices [5]. Chemically synthesized agrochemicals, such as fungicides and pesticides, are sprayed to prevent microbial crop diseases. But these chemicals are quite hazardous to crops and are potentially toxic to human health [6]. In most cases, agrochemicals applied in fields are unable to reach the targeted sites due to factors like leaching, hydrolysis, photolysis, and especially microbial degradation [7]. Due to nano size, nanofertilizers and nano pesticides can be easily distributed in a very controlled fashion with precise specificity, thereby reducing collateral damage. Nanotechnological applications in farming have gained attention because of their well-organized control and accurate release of herbicides, pesticides, and fertilizer [8]. The role of agrochemicals is crucial in modern agriculture, but the development of nanofertilizers and nano pesticides transformed the agricultural sector [9].

Zinc is a vital micronutrient required for all living organisms, and its deficiency in humans is considered a cause of malnutrition worldwide [10]. Zinc is considered second most important transition element in living organisms after iron and is present in all six enzymes classes (ligases, hydrolases, isomerases, transferases, oxidoreductases, and lyases) [11]. Zinc nanoparticles are used widely in many fields, e.g., agriculture and medicine. Zinc nanoparticles have been reported to influence plant growth, yield, and fatty acid profiles in diverse plant species [12]. Increased germination rates and root length in maize plants were noticed after exposure to zinc oxide nanoparticles [13], whereas increased shoot length was observed in oat and berseem plants [14]. ZnO nanoparticle application has meaningfully augmented the chlorophyll content, plant height, and plant biomass of tomato plants [15]. Zinc nanoparticles also enhanced plant growth and increased root biomass of the *Glycine max* plant [16]. Foliar application of zinc oxide nanoparticles significantly increased the leaf area and dry mass by 69.7% and 63.8%, in maize respectively [12]. The applications of zinc nanoparticles showed a promising upsurge in leaf area, plant height and yield of strawberry plants [17].

Canola (*B*. *napus*) is a cool-season, broadleaf member of the *Brassicaceae* family. Rapeseed has been cultivated in Asia for centuries and in Europe since the 13^th^ century. Canola cultivars were developed using traditional breeding methods in Canada. Canola has been regarded as harmless by the United States of Food and Drug Administration since 1985 [18]. Canola oil are reported to have less than 2% of erucic and 30 μmole of glucosinolate per gram of seed meal. Due to low saturated and polyunsaturated fatty-acid, canola oil is a good replacement for hydrogenated soybean oil in the cooking industry. *B*. *napus* is also known as canola, Rapa, rappi [19]. *B*. *napus* is an important crop due to the production of oil and is considered the third major source of edible oil after palm and soya. The oil is used for medicinal and food purposes, while plant parts are also used as traditional medicine [20]. Roots of *B*. *napus* are widely used in the therapeutic treatment of many diseases, as it has a diuretic, anti-scurvy, anti-gout, and anti-inflammatory effect on bladder infections. The seeds of this plant have been used to treat hepatic, kidney, and bronchial cathartic problems [21].

Considering the importance of *B*. *napus* and nanotechnological applications in the agricultural industry, the current project was designed to evaluate the effects of green synthesized zinc nanoparticles (Zn NPs) on biochemical profiling, antioxidant enzymes, and the nutritional and fatty acid profile of *B*. *napus* varieties. To the best of our knowledge, this is the first study to examine the effects of Zn NP’s on *B*. *napus* from Pakistan.

## Materials and methods

### Preparation of leaf extract and green synthesis of nanoparticle

Approximately 20 g of commercially available *M*. *arvensis* leaves were cut into small pieces with scissors, immersed in 100 ml of Milli-Q water, and placed on a hotplate at 50°C for 4 hours. The reaction mixture was allowed to cool at room temperature. The cooled mixture was then filtered twice with Whatman 21 filter paper, and the filtrates were stored in a refrigerator at 4°C, according to the procedures outlined by Dobrucka and Długaszewska [22]. The green synthesis was carried out by reduction of Zn (NO_3_)_2_.6H_2_O with the Mentha plant extracts. In this process, 1.2939 mg of zinc nitrate heptahydrate was dissolved in 900 ml of Milli-Q water and allowed to stir with a magnetic stirrer while continuously drop wise addition of 100 ml of Mentha extract was done. This took 40 minutes and a light brown colour appeared, which indicated the synthesis of the Zn NPs. The solution was then subjected to KUBOTA centrifuge Model 2420 at 10000 x g, and the pellet was collected, and washed with ethanol twice to remove any impurities. The collected pellet was kept in the oven at 100°C for drying and then transferred to airtight vial [23].

#### Characterization of nanoparticles

Green synthezised nanoparticles were subjected to different characterization techniques like, Ultraviolet visible spectroscopy (UV) Transmission electron microscope (TEM) Center for Molecular Biology of Plants, Eberhard Karl’s University of Tuebingen, Germany, X-Ray Diffraction (XRD) National Center of Physics, Quaid e Azam University Islamabad, Energy dispersive X-ray (EDX), Scanning Electron Microscope (SEM) Institute of Space Technology, Islamabad Pakistan [24].

### Plant material and growth condition

The greenhouse experiment was carried out using soil pots. The standard pot size diameter of 25 cm and height 24 cm was used. Seeds of rapeseed (*B*. *napus*) varieties were obtained from the National Agriculture Research Center Islamabad, Pakistan. Locally available two different varieties of rapeseed (*B*. *napus*) varieties Faisal canola and Shiralee were used in the experiment. Seeds treated with Zn NPs and without were directly sown into soil-filled pots and kept in a greenhouse at 22°C with a light density of approximately 120 mol m-2s-1, followed by dark photoperiod of 16 h/8 h. Seed was then germinated and grown in soil pots. Soil analysis was carried out in the Institute of soil sciences PMAS UAAR, Rawalpindi, Pakistan. Soil analysis showed that the pH of the soil was 7.48 having a loamy texture. Total organic matter recorded in the soil sample was 0.80%, available nitrogen 0.35%, phosphorus 5.5mg/kg and potassium were 100mg/kg. The EC of the tested soil recorded was 1.74 dsm^-1^, however, soil was zinc deficient having the total zinc of 0.92 mg/kg.

#### Zn NPs treatment

Three different concentration of Zn NPs 5 mg/L, 15 mg/L and 25 mg/L were prepared and applied in three different ways, i.e. treated seed (5mg/L, 15mg/L, 25mg/L), foliar spray (5mg/L, 15mg/L, 25mg/L), and combination of treated seed and foliar spray (5mg/L, 15mg/L, 25mg/L) Table 2. In the first experiment, rapeseed seeds were soaked in the above mentioned concentration of Zn NPs solution for 20 min and were sown directly in soil-filled pots. In the 2nd experiment, 2 consecutive foliar sprays of Zn NPs 5 mg/L, 15 mg/L, and 25 mg/L was made after the appearance of first secondary leaves followed by 2nd foliar spray at the flowering stage. Plants in each pot were sprayed with 20 ml of Zn NPs of the indicated concentrations. In the 3rd experiment the combined effect of Zn NPs treated seed and foliar spray was investigated. After germination, the extra seedling was removed to ensure that every pot contains only three plants. All the plants were watered regularly till five (5) weeks and after that plants were watered twice a week. Fresh and healthy leaves from fifty-five days old plant were collected for biochemical, antioxidant enzymes analysis, and the plants were left to complete its life cycle to ensure seeds formation. After that plants were harvested, and seeds were collected to investigate grain quality parameter and fatty acid profiling. Completely Randomized Design was followed, and three different individual biological replicates were used in the study as profiled in Table 2.

### Biochemical profiling

#### Protein content

For the determination of the protein content method of Bates et al. [25] was followed. Fresh leaf material (0.2 g) was ground in 10ml of phosphate buffer in a tube followed by filtration. About 0.5 ml of the filtered extract was mixed with 0.5 ml of distilled water and 3 ml of bio-red dye (25 times diluted). The mixture was incubated for 30 min at room temperature and absorbance was noted at 595 nm.

#### Total amino acid (TAA)

Amino acid analysis of samples was based on methodology previously reported [23]. Plant leaf material, about 0.2 g was powdered in 10 ml of phosphate buffer and filtered. One ml of filtered extract was mixed with 1ml of pyridine solution and 1 ml of ninhydrin solution. The reaction mixture was boiled at 100°C for 30 mins and absorbance was noted at 570 nm using spectrophotometry model Cecil 2021.

#### Proline content

To assess proline content, fresh leaf material of 0.5 g was ground with 3% of sulfosalicylic acid in a test tube and the solution was kept for 30 min to settle down. About 2ml of the supernatant extracts were mixed with 2ml of glacial acetic acid and 2 ml of ninhydrin reagent. The mixture was then boiled in a water bath at 100°C for 1 hour. The reaction was stopped on ice and finally, 4 ml toluene to was added. The mixture was shaken well, and the upper layer was collected to new test tubes. The absorbance was noted at 520 nm [25].

#### Total soluble sugar (TSS)

For the determination and quantification of total soluble sugar, 0.2 g of fresh leaf material was mashed in 10ml of 80% ethanol and filtered. The filtered extract of 0.5ml was mixed with 0.5 ml of distilled water and 1 ml of 18% phenol solution. The mixture was kept at room temperature for 1 h, afterwards 2.5 ml of sulphuric acid was added followed by shaking for 30 min. The absorbance of the samples was noted at 490 nm using Cecil 2021 spectrophotometry [26].

#### Total phenolics content (TPC)

Folin-ciocalteu reagent was used for the quantification of total phenolics contents in the extracts, following the method described by Fu et al. [27]. About 0.2 g powdered plant material was crushed with 80% ethanol. The reaction mixture was prepared by mixing 100 μl of plant extract, 0.75 ml of folin-ciocalteu reagent, and was incubated at 21°C for 10 min. The reaction mixture was equipped with 0.75 ml of sodium bicarbonate solution followed by incubation at 21°C for 90 min. The absorbance of the samples was recorded at 725 nm, using the Cecil UV-visible spectrophotometer.

#### Total flavonoid content (TFC)

The aluminum chloride colorimetric method was used for the determination of the total flavonoid content of the sample [28]. Ten grams of quercetin was dissolved in 80% ethanol and further dilutions were prepared to plot the standard curve. The reaction mixture contains plant extract, 1.5 ml of ethanol, 1M CH_3_CO_2_K, 2.5 ml of water, and 0.2 ml aluminum chloride AlCl_3_. This reaction mixture was kept at room temperature for 60 min. Using the Cecil UV-visible spectrophotometer, the absorbance of the reaction mixture was recorded by setting standard wavelength of 415 nm.

### Antioxidant enzymes

#### Catalase activity (CAT)

Catalase activity was measured by the disappearing of H_2_O_2_. The reaction mixture contained 20μl of enzymes extract, 50mM of potassium phosphate buffer (pH 6.8), 1mM EDTA, and 15mM H_2_O_2_. The activity of CAT was measured by the decrease in H_2_O_2_ in 1 min at the absorbance of 240 nm. CAT activity was calculated by its extinction coefficient of 6.93 mM^-1^ cm^-1^ [29].

#### Peroxidase (POD)

Peroxidase activity was determined by using the method described by [30] with a slight modification. The 1ml reaction mixture contained 5 mM H_2_O_2_, 15 mM guaiacol, and 40mMphosphate buffer (pH 6.8). The reaction mixture was kept for some time and then the reaction was initiated by adding H_2_O_2_ while the increase in the absorbance at 470 nm was recorded for 1 min. Measurement of POD activity was carried out as per its extinction coefficient of 25 mM^-1^ cm^-1^.

#### Superoxide dismutase (SOD)

To measure superoxide dismutase approximately ten ml of prechilled sodium phosphate buffer was used to grind 0.5g leaf material. The mixture was centrifuged for 15 min at 10000 x g at set 4°C. On settling down of solution, a separate set of test tubes containing 0.1ml of the plant extracts were supplemented with 0.1 ml riboflavin and 3ml of SOD buffer. This reaction mixture was placed under the fluorescent lamp for 8 min to start the reaction. The same reaction mixture was prepared for dark reaction in another set of test tubes. The absorbance was noted at 560 nm wavelength [23].

### Nutritional and elemental analysis

#### Zinc, sodium, and potassium content

Zinc, sodium, and potassium content were assisted using the methodology layout by Allen et al. [31]. For this purpose, 0.5g of dried powdered leaf material was digested in nitric acid and perchloric acid with a ratio of 2:1 for 6 h resulting in a clear and colorless solution. The volume of the sample was adjusted to 25 ml by the addition of distilled water. The standard solutions i.e. 10, 50, 100, and 200mg/L of Zn (NO_3_)_2_ were prepared. The standard curve was plotted and zinc content in the plant was standardized with the standard curve. Zinc concentration in the sample was measured through Perkin Elmer atomic absorption spectroscopy Model 460, while sodium and potassium content concentration were determined using Digital flame photometer Model fp-2228.

### Grain quality parameters and fatty acid profiling

Plants were harvested and grain quality parameters including grain moisture, grain protein, oil contents and fatty acid profile were determined by Near-Infrared Reflectance Spectroscopy in the Nuclear Institute for Food and Agriculture, Peshawar, Pakistan using the protocol in [32].

### Statistical analysis

All the experiments were performed in triplicates, data were analyzed statistically through analysis of variance (ANOVA) and mean significant differences were separated using Duncan’s Multiple Range Test (DMRT).

### Ethical statement

This material is the authors' own original work, which has not been previously published elsewhere. This study does not involve any human or animal trials and was purely based on use of green synthesized zinc nanoparticles for the growth and development of rapeseed plant.

## Results and discussion

### Synthesis and characterization of Zn nanoparticles

Nanotechnology is widely used in several fields of science and mainly concerned with the synthesis of NPs of different sizes, shapes, and chemical nature [33]. Green synthesis of nanoparticle was carried out using *M*. *arvensis* plant extract and Zn (NO_3_)_2_.6H_2_O with a 9:1 ratio. The solution of synthesized zinc nanoparticle was light brown in color indicating the presence of Zn NPs. A number of extracts from different plants were successfully used for the synthesis of Zn nanoparticles such as *Albizia saman* [34], *Nyctanthes* flower, *Garcinia mangostana* [35, 36] and *Ulva Lactuca* [37]. After green synthesis of Zinc nanoparticles, different characterization techniques were used to study the physicochemical properties of the synthesized nanoparticle i.e. (UV-vis) spectroscopy, scanning electron microscopy (SEM), transmission electron microscopy (TEM), X-Ray diffraction (XRD), energy dispersive X-Ray (EDX) and particle size distribution was done by using ImageJ software. These physical properties play a vital role in the novel application of nanoparticles. Redundant researchers reported several plants extracts for the synthesis of metallic NPs [23, 37].

#### Uv-visible spectroscopy

**Fig 1:**
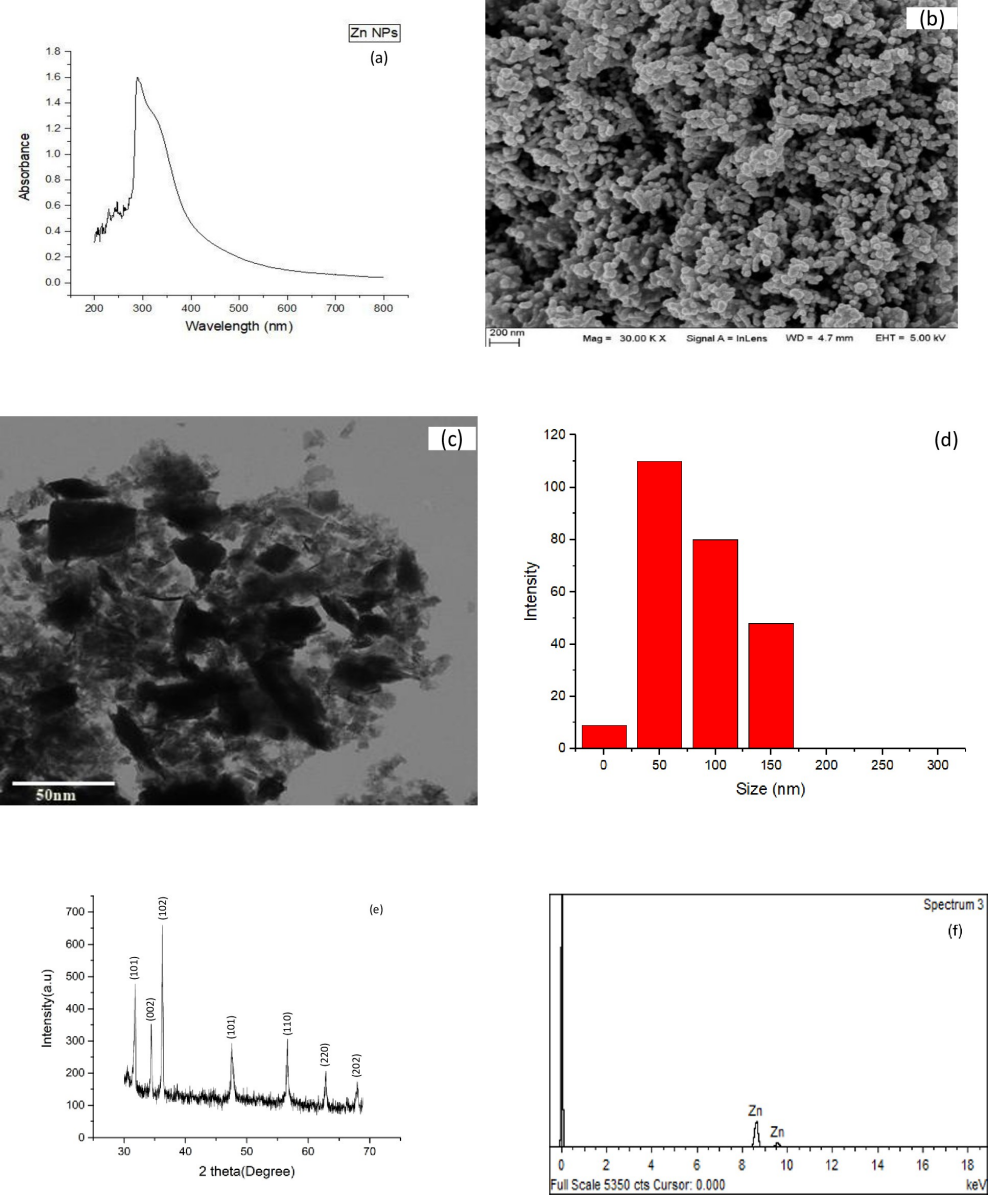
Nanoparticle characterizations using different techniques like (a) Uv-visible spectrum, (b) Scanning electron microscopy, (c) Transmission electron microscopy, (d) Size Distribution, (e) X-Ray Diffraction (XRD), (f) Energy Dispersive X-Ray (EDX).

UV-visible spectroscopy is based on measuring the absorbance of a substance within a certain wavelength spectrum. It is an important technique widely used to confirm the desired substance in the sample. In order to confirm the synthesis of zinc nanoparticles UV-visible spectroscopy Beckman Model DU640 was used. The synthesized nanoparticles were dispersed using ultra probe sonicator Model 750 for 30 min. The UV-vis spectroscopy results confirmed the synthesis of zinc nanoparticle by indicating a sharp peak at 292 nm Fig 1A. [23, 38]. This sharp peak exhibits the monodispersed nature of green synthesized Zn NPs. This peak results from the absorption of the visible light by the electron present in the zinc element.

#### SEM and TEM analysis

Scanning electron microscopic analysis was done by using the SEM SIGMA model (MIRA3; TESCAN Brno) at the Institute of space technology Islamabad, Pakistan. The SEM revealed the irregular shape and morphology of green synthesized Zn NPs, previously published by many researchers [34, 36]. Transmission electron microscopy was carried out at the Center for Plant Molecular Biology, University of Tuebingen, Germany. Different magnifications lenses were used to study the morphology and size of NPs. The copper grid was used to study the morphology of a single nanoparticle. The TEM results confirmed that nanoparticles were irregular in shape authenticating the results of the scanning electron microscope. The particle size distribution was carried out using ImageJ software. The average size range of nanoparticle was 30–70 nm which is considered good for its optimum activity Fig 1B–1D.

#### X-ray diffraction (XRD)

This technique authenticates the crystalline nature of nanoparticles and provides the average size of the particle by using Debye Scherrer’s equation. The definite sharp bordering line of XRD peaks indicates the presence of zinc in nano form in the sample. In the current study, XRD analysis of the synthesized Zn NPs revealed the confirmation of zinc nanoparticle by comparing with the joint committee on powdered diffraction standard number and the average size range was 30–70 nm [39]. Form this XRD pattern we can determine peak intensity, width, position, full width, and half maximum (FWHM) Fig 1E. The sharp peaks from the XRD were observed at 31.5°, 34.6°, 37.6°, 47.8°, 57.6°, 63.7°, 68.4° degree showing the hexagonal phase of green synthesized Zn NPs.

#### Energy dispersive X-ray (EDX)

Elemental analysis of synthesized NPs is of immense importance and plays a vital role in the novel properties of nanoparticles. To confirm the purity of synthesized NPs, the elemental composition of synthesized nanoparticle was assisted using an energy dispersive X-ray technique. In the current study, EDX results confirmed that the zinc percentage in the sample was 98.34%, Al (0.4%), and O (1.23%) with the atomic weights of 94.20, 1.00, and 4.81 respectively Table 1 and Fig 1F. This technique confirms the purity of nanoparticles by showing the percentage of elements in the sample by atomic weight and weight in percentage.

**Table 1:** Elemental composition from EDX of green synthesized zinc nanoparticle.

Element	Weight%	Atomic%
**O K**	1.23	4.80
**Al K**	0.43	1.00
**Zn K**	98.34	94.20
**Total**	100.00	100.00

### Biochemical profiling

#### Protein content

**Fig 2:**
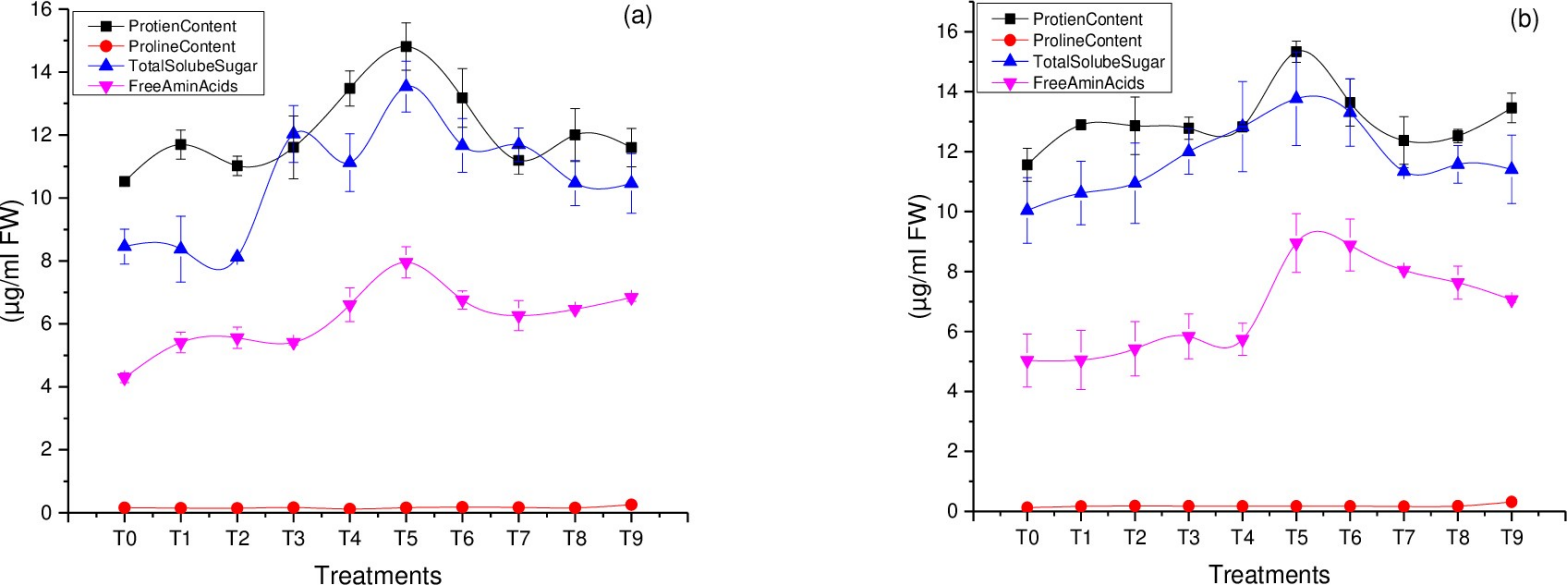
Different biochemical traits characterization of rapeseed (*Brassica napus*) varieties as influences by green synthesized Zn NPs. The data is profiled in two different graphs for each variety (a) indicates rapeseed variety Faisal canola and (b) indicates Shiralee. The data in the graphs were expressed in means ± SD, keeping P value <0.05.

The importance of Zn in plant growth development, reproduction, and yield is non-negligible [40]. Among all the Zn nanoparticle treatments, the T5 (foliar treatment of the Zn nanoparticles 15 mg/L) was significantly higher as compared to Zn nanoparticles treated seeds. In the current study, exposure to T5 (foliar treatment of the Zn nanoparticles 15 mg/L) caused a note-worthy increase in protein content of canola varieties, Faisal canola, and Shiralee (40%) and (32%) respectively Fig 2A and 2B. Earlier reports suggested that biosynthesized NPs interact with meristem cells triggering different biochemical pathways [41]. Many researchers have stated a significant upheaval in certain biochemical aspects such as, glutelin (1.6 times), starch content (2 times), and Zn content (2.5 times) in cucumber plant on exposure to ZnO NPs. This increase in protein content might be a metabolic balance between the induction of proteins and micronutrients like zinc. The role of zinc is crucial in numerous biochemical reactions inside plants i.e. chlorophyll and carbohydrates [42, 43].

#### Free amino acid content (FAA)

Amino acids play a vital role in the synthesis of proteins and only L-amino acid contributes to plant metabolic protein. The free amino acids were determined in plants treated with and without Zn NPs. Free amino acid content showed a noteworthy difference among canola varieties. Exposure to the T5 (foliar treatment of the Zn nanoparticles 15 mg/L) increased the free amino acid of both canola varieties (85% for the Faisal canola and 77% for the Shiralee) Fig 2A and 2B. Mesoporous silica NPs increase sugar content in wheat [44]. Plants growing in zinc-deficient environments have decreased the number of growth regulators, short internodes, and curly leaves. Zn is the most crucial factor of many enzymes, structural proteins, membranes, and DNA binding proteins [42]. However, Zn has been confirmed as the safest substance by the United States Food and Drug Administration [12].

#### Total soluble sugar content (TSS)

Sugar, proline, and SOD are considered to play a crucial role in plant defense mechanisms [42]. The sugar content was significantly higher (60%) for Faisal canola and (37%) for Shiralee following exposure to T5 (foliar treatment of the Zn nanoparticles 15 mg/L). However, in relation to T1 (treated seed, 5 mg/L) and T9 (combination of treated seed + foliar spray of 25mg/L Zn nanoparticles) slightly increase the sugar content of the canola varieties, which was (5%) for Faisal canola and (23%) for canola variety Shiralee respectively Fig 2A and 2B. Our results are in agreement with [45] who stated increase sugar contents in rice seedling on exposure to ZnO NPs application.

#### Proline content (PC)

Proline content is usually produced under stress conditions and plays a key role in protecting protein denaturation thus leading to maintain normal cellular metabolism [46]. It can be seen that both the canola varieties exhibited significantly higher proline content at T7 Zn nanoparticles (5 mg/L) T8 (15 mg/L) and T9 (25 mg/L combination of treated seed + foliar spray). The proline content was higher at T9 (combination of treated seed + and foliar spray of 25mg/L Zn nanoparticles) caused a remarkable increase of (35% and (45%) for Faisal canola and Shiralee respectively when compared to control plants Fig 2A and 2B. Proline is an important adjustor for stress relieving like metal stress, scavenging radicles and it maintains water balance in such conditions. However, no such reduction in proline content was observed on exposure to green synthesized Zn NPs. The positive role of proline in mitigating lipid peroxidation was demonstrated in *Chlorella vulgaris* under metal stress conditions [47].

#### Total flavonoid content (TFC) and total phenolics content (TPC)

**Fig 3:**
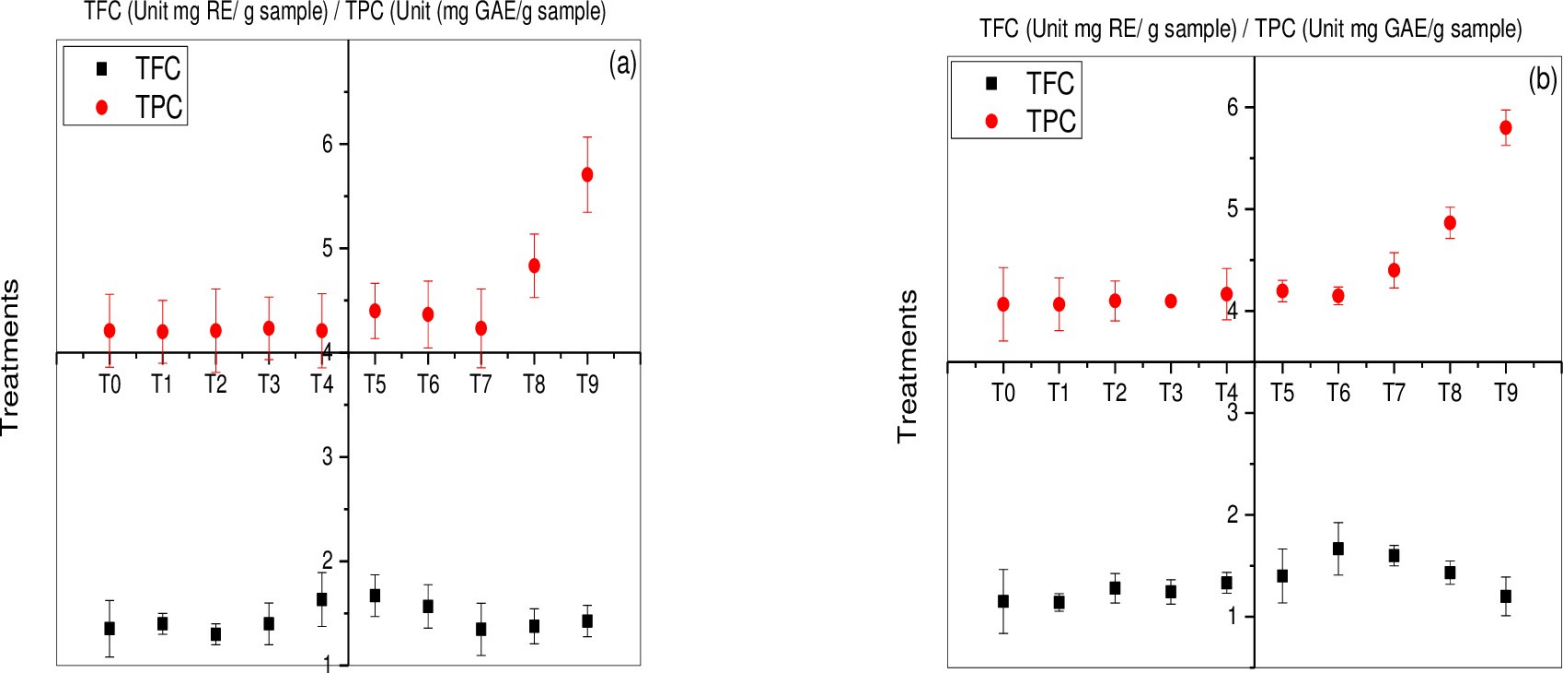
Total flavonoid content and total phenolic content in rapeseed (*Brassica napus*) varieties under varying stress of green synthesized Zn NPs. The data is profiled in two different graphs for each variety (a) indicates rapeseed variety Faisal canola and (b) indicates Shiralee. Values are means of three biological replicates (±SD)., while P value was kept <0.05.

Flavonoids are one of the essential bioactive chemical compounds produced in plants. It plays a vital role in the scavenging of free radicles by working as an antioxidant [48]. It has been observed that flavonoid content was significantly increased at T5 and T6 (15mg/L and 25 mg/L foliar spray Zn nanoparticles) causing an increase of (23%) and (44%) in the canola varieties Faisal canola and Shiralee, respectively. However, a slight increase was observed at T1 and T2 (Zn NPs 5mg/L and 15mg/L) treated seed causing an increase of (3%) and (11%) for Faisal canola and Shiralee, when compared to plants in control condition Fig 3A and 3B. Our results are in agreement with [49] who reported increased flavonoid content in the floral leaf of broccoli.

Zinc plays a vital role in the management of oxidative stress and reactive oxygen species protecting plant cells [50, 51]. Such exposure to Zn NPs application significantly improved the production of flavonoid content in *B*. *napus* varieties. On the other hand, maximum phenolic content was shown by T9 (Zn NPs 25mg/L combination of treated seed and foliar) causing an increase of (35%) and (42%) in canola varieties Faisal canola and Shiralee individually Fig 3A and 3B. Abiotic stresses could cause significant production of phenolics contents in plant species [52]. In general, little changes in phenolics content of canola varieties Faisal canola and Shiralee was observed on exposure to Zn NPs.

### Antioxidant enzymes

#### Superoxide dismutase (SOD), peroxidase activity (POD), and catalase activity (CAT)

**Fig 4:**
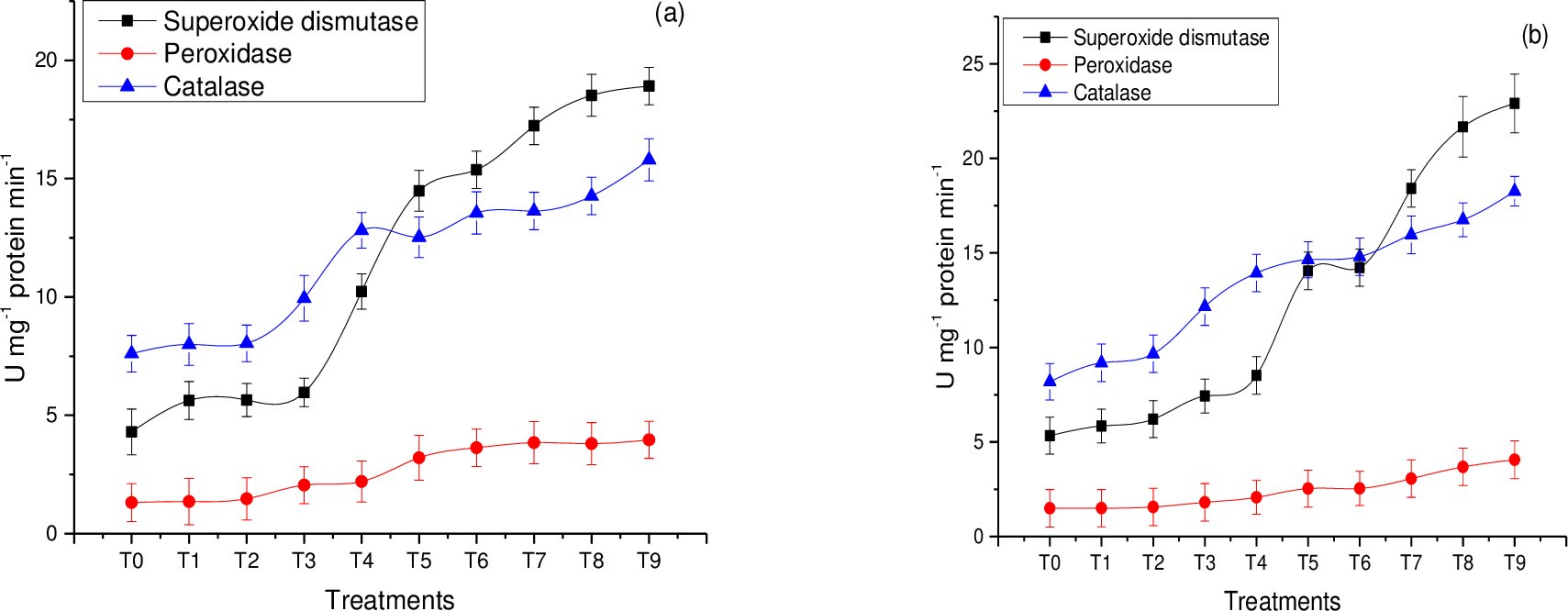
Antioxidant enzyme activities of rapeseed (*Brassica napus*) varieties under varying stress of green synthesized Zn NPs (a) Faisal Canola and (b) Shiralee, respectively. The data in the graphs were expressed in term of means ± SD, while P value was kept <0.05.

Antioxidant enzymes have a vital role in mitigating the adverse effects of ROS species on photosynthesis and photorespiration. Superoxide dismutase is the first-line defense against oxidative stressors, and it catalyzes H_2_O_2_ into water and molecular oxygen species by modulating the amount of hydrogen peroxide and oxygen [53]. In the current study, the dose-dependent tendency was observed, as the concentration of NPs increased, the increased activities was recorded. In the current study, it can be seen from the results that superoxide dismutase activity was significantly higher at T9 (25 mg/L combination of seed treated + foliar spray) which was (22.9 U mg^-1^ protein min^-1^) for Shiralee and (18.9 U mg^-1^ protein min^-1^) for Faisal canola respectively Fig 4A and 4B. High SOD activity in *Arabidopsis thaliana* was measured on exposure to Cu NPs [54] confirms our results.

Whereas, the peroxidase activity was maximum at T9 (25 mg/L Zn nanoparticles combination of treated seed + foliar spray) which was (4.06 U mg^-1^ protein min^-1^) for canola variety Shiralee and (3.9 U mg^-1^ protein min^-1^) for Faisal canola when compared to the plants in control condition Fig 4A and 4B. This increase in growth parameters may be due to the release of more Zn^2+^ ions for plant development. Increased antioxidant activities certainly exposed that NPs could probably trigger the peroxidase activity which directly leads to overcome different stresses. Zinc nanoparticles are responsible for the increased activity of SOD and POD in different crop plants such as cucumber, Alfalfa, and tomato [55].

Catalase activity showed a significant difference among *B*. *napus* varieties i.e. (Faisal canola and Shiralee). The highest catalase activity was observed in plants treated with T9 (25mg/L combination of treated seed and foliar spray), which was (18.2 U mg^-1^ protein min^-1^) for canola variety Shiralee and (15.2 U mg^-1^ protein min^-1^) for Faisal canola. Whereas, the lowest peroxidase activity was observed in (5mg/L treated seed) (12 U mg^-1^ protein min^-1^) in canola variety Shiralee and (5 U mg^-1^ protein min^-1^) in Faisal canola respectively Fig 4A and 4B. Similarly, constructive reports were published by [56] leading to improved growth parameters at a low concentration of ZnO NPs.

### Nutritional and elemental analysis

#### Zinc (Zn), sodium content (Na+) and potassium content

**Fig 5:**
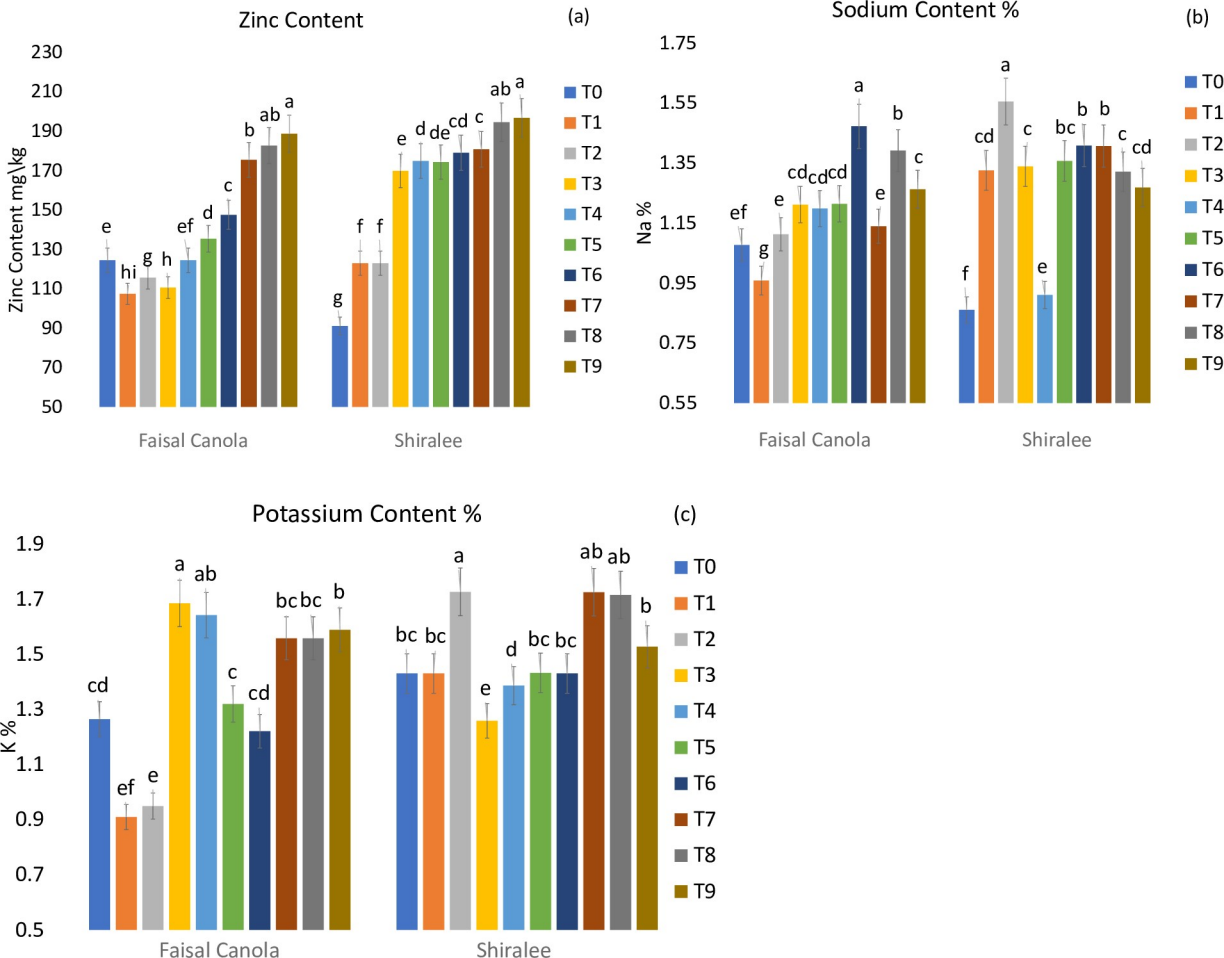
The effect of green synthesized Zn NPs on the nutritional and elemental profile (a) Zinc contents, (b) Sodium contents and (c) Potassium contents of tested Rapeseed Varieties Faisal canola and Shiralee. All the Values are the means of three biological replicates (Mean ± SD).

Zinc has an essential role in protein and carbohydrates; it also controls different growth hormones and has a vital role in crucial components of peptides, enzymes, proteinase, dehydrogenase and starch development, seed production, ripening and production [57]. In the current report, leaf zinc, sodium, and potassium content of *B*. *napus* leaves were determined after exposure to Zn NPs. The maximum zinc contents in leaves of canola varieties were recorded at T9 25 mg/L Zn NPs combination of treated seeds with a foliar spray: 196.7 mg/kg in Shiralee and 188.7 mg/kg in Faisal canola Fig 5A. As shown in Fig 4. The Zn contents were higher in different parts of *Vigna unguiculate* [58] while significant Ce and Zn accumulation was recorded in various parts of soybean on exposure to ZnO NPs and CeO NPs [59]. NPs accumulation in plants is a matter of concern as it may lead to detrimental environmental effects. On the other hand, exposure to Zn NP application consistently and significantly improved Na+ content in canola varieties Faisal canola and Shiralee.

The maximum leaf Na^+^ content for Faisal canola was 1.47% at T6, with Zn NPs 25 mg/L foliar spray, and the maximum for Shiralee was 1.55% at T2 with Zn NPs 15 mg/L treated seed Fig 5B. However, significant potassium content was found in both varieties when treated with T2: 15 mg/L Zn NPs treated seeds were 1.68% and 1.72% for Faisal Canola and Shiralee, respectively Fig 5C. The roles of different membrane transporters in the uptake of micronutrients like P, Cu, Fe, and Zn are well known but the mechanism behind the entry of metallic NPs in plant cells and the ways they trigger different signaling pathways are poorly understood. Nanoparticles can have either a detrimental or beneficial effect on plant agronomical and physiological traits, including the nutritional value of food crops [60].

### Grain quality parameter

Grain of *B*. *napus* were analyzed using the Near-Infrared Reflectance Spectroscopy model 6500, Foss NIR Systems Inc., MD, USA) at NIFA [Nuclear Institute of Food and Agriculture]. Details about this procedure have already been published in [61]. It can be seen that both the canola varieties exhibited a dramatic decrease in oil content on exposure to such Zn nanoparticles applications. The oil content was higher in control plants which were (46.6%) for Faisal canola and (41.1%) for Shiralee, respectively. However, oil content in Zn nanoparticles treated plants were (38.6, 38.9, 39.4,38.4, 42.8, 38.9, 39.6, 38.6, 39.6) for Faisal canola and (40.1, 37.6, 40.2, 40.2, 40.1, 37.5, 38.2, 37.2, 38.1) for Shiralee at T1, T2, T3, T4, T5, T6, T7, T8, and T9, respectively. To our knowledge, this is the first study to investigate the effect of metallic nanoparticles on the oil content of *B*. *napus* fatty acid, as profiled in Table 2.

**Table 2:** Effect of Zn NPs exposure on seed quality parameters and fatty acid profile of rapeseed (*B*. *napus*) varieties Faisal canola and Shiralee.

Treatments	Seed Quality Parameters						Fatty acid profile								
	Oil Content %		Protein Content %		Moisture Content %		Oleic Acid % (18:1)		Linolenic Acid % (18:3)		Erucic acid % (C22:1)		GSL μmol/g	
Varieties	V-1	V-2	V-1	V-2	V-1	V-2	V-1	V-2	V-1	V-2	V-1	V-2	V-1	V-2
**T0**	46.6 ± 1.3 a	41.1± 1.21a	24.8 ± 1.6 d	28.1 ±1.2 d	10 ± 0.97 a	8.6 ± 0.67a	53.6 ± 2.56 g	59.5 ± 1.34 df	9.5 ±1.20 c	10.6 ±1.02 a	1.5 ± 0.65 b	1.2 ± 0.99 f	27.4 ±0.97 a	27.6 ± 2.20 bc
**T1**	38.6± 1.5 cd	40.1± 0.98 a	29.4 ± 1.45 b	31 ± 2.1 ab	8.1 ± 0.87 c	8.1 ± 0.89 b	64.4 ± 2.41 c	61.2 ± 1.25 c	11 ± 1.00 b	7.8 ± 0.99 c	1.4 ±0.69 c	1.3 ± 0.96 d	24.5 ± 2.5 c	29.9 ± 0.98 a
**T2**	38.9 ± 1.02 cd	37.6 ± 0.89 c	29.2 ± 1.43 b	31.1 ± 1.2 ab	8.4 ± 0.99 bc	8.1 ± 1.00 b	64.2 ± 1.46 c	59.6 ± 1.25 df	11.6 ± 1.35 ab	7.8 ± 1.25 c	1.7 ± 0.98 a	1.4 ± 0.86 c	24.7 ± 0.97 c	28.5 ± 1.43 b
**T3**	39.4± 1.23 c	40.2 ± 0.98 a	29.8 ± 1.33 ab	30.7 ± 2.2 bc	8.1 ± 0.89 c	8.1 ± 1.76 b	66.4 ±1.98 b	61 ± 1.56 cd	12.6 ± 1.25 a	8.9 ± 0.56 b	1.3 ± .96 d	1.3 ± 0.82 d	25.6 ±b	28.8 ± 0.97 b
**T4**	38.4± 1.25 cd	40.2 ± 0.99 a	30.8 ± 0.98 a	30.2 ± 1.45 c	8.6 ± 0.76 bc	7.9 ± 0.78 bc	58.5 ± 1.87 e	66.1 ± 2.68 a	9.1 ± 1.34 c	7.6 ± 0.89 c	1.5 ± 0.96 a	1.6 ± 0.99 b	23.4 ± 0.97 d	28.7 ± 0.98 b
**T5**	42.8± 1.78 b	40.1 ± 1.09 a	28.7± 0.94 c	31.3 ± 1.87 ab	9 ± 0.98 b	8.3 ± 0.91 b	57.9 ± 1.67 ef	59.7 ± 2.98 e	9.6 ± 0.99 c	9.2 ± 1.2 b	1.4 ± 0.91 c	1.4 ± 0.91 c	23.5 ± 2.2 cd	29.8 ± 2.2 a
**T6**	39.5± 1.68 c	37.5± 2.50 c	29.2 ± 1.35 b	31.6 ± 1.35 a	8.4 ±0.99 bc	8.1 ± 0.99 b	62.4 ±2.50 bc	61.5 ±1.87 cd	9.8 ± 0.99c	8.4 ± 0.98 cb	1.3 ± 0.96 d	1.3 ± 0.65 d	23.8 ± 0.99cd	27.4 ± 11.1bc
**T7**	39.6 ±1.98 c	38.2 ± 2.18 b	29.5 ± 1.23 b	30 ± 1.38 c	7.8 ± 1.00 cd	8.5 ± 0.99 a	62.9 ± 2.65 bc	61 ± 1.35 d	12 ± 1.25 a	9.4 ± 1.00 a	1.3 ± 0.87 d	1.4 ± 0.68 c	23.6 ± 1.25 cd	27.4 ± 1.35 bc
**T8**	38.6 ± 1.87 cd	37.2 ± 2.00 c	30 ± 1.45 a	32 ± 1.38 a	7.8 ± 0.98 cd	8.2 ± 0.87 b	61.3 ±1.98 d	60 ± 1.87 d	11.7 ± 1.54 ab	8.5 ± 0.68 cb	1.4 ± 0.58 c	1.4 ± 0.57 c	25.7 ± 1.47 b	24.3 ±1.00 d
**T9**	39.6 ± 0.99 c	38.1± 1.25 b	30.2 ± 1.87 a	30.9 ± 1.37 bc	8.2 ± 0.58 c	7.4 ± 0.88 e	68.4 ± 199 a	64.6 ± 1.36 b	11.7 ± 0.99 ab	7.5 ± 1.5 c	1.5 ± 0.24 b	1.7 ± 0.57 a	23.7 ± 1.11 cd	27.8 ± 1.09 bc

Varieties: **V-1** (Faisal Canola) **V-2** (Shiralee), **T0** (Control), **T1** (Treated Seed with 5mg/L), **T2** (Treated Seed with 15mg/L), **T3** (Treated Seed with 25mg/L), **T4** (Foliar Spray with 5mg/L), **T5** (Foliar Spray with 15mg/L), **T6** (Foliar Spray with 25mg/L), **T7** (Treated Seed with 5mg/L + Foliar Spray with 5mg/L), **T8** (Treated Seed with 15mg/L + Foliar Spray with 15mg/L), **T9** (Treated Seed with 25mg/L + Foliar Spray with 25mg/L). The data presented in columns in the form of means having similar letter are identical and dissimilar letter are significantly different where P Value was kept P <0.05.

Canola protein and its nutritional quality could be a good source of suitable food ingredients for human consumption [62]. In the current study, the maximum protein content in grains of canola varieties was 31.6%, observed at Zn NPs T7 (25 mg/L foliar spray) in Shiralee; the next highest was 30.8% at T5 Zn NPs (5 mg/L foliar spray) in Faisal canola Table 2. With exposure to Zn NPs foliar application, protein contents in *B*. *napus* varieties Faisal canola and Shiralee were improved.

Grain moisture content is an important parameter of oilseed crops, as it is directly proportional to the life span of seeds [63]. The highest moisture content was found in the Faisal canola variety (10%) followed by Shiralee (8.6%) under control conditions. Reduced moisture content was found in treated plants (8.1%, 8.4%, 8.1%, 8.6%, 9%, 8.4%, 7.8%, 7.8% and 8.2% in Faisal canola and 8.1%, 8.1%, 8.1%, 7.9%, 8.3%, 8.1%, 8.5%, 8.2% and 7.4% in Shiralee at T1, T2, T3, T4, T5, T6, T7, T8 and T9, respectively) Table 2. This decrease in moisture content could result from the higher concentration of the Zn NPs. The effect of nanoparticle treatment depends on the size, shape, structure, and concentration of nanoparticles and varies from plant to plant. Zn is generally a mobile element in plant tissue and various researchers have reported on the importance of Zn in plant growth development, reproduction, and yield. Micronutrients like zinc perform a crucial role in numerous biochemical reactions inside plants (i.e. chlorophyll and carbohydrates) [42]. Earlier reports have suggested that biosynthesized NPs interact with meristem cells to trigger different biochemical pathways [41].

### Fatty acid profiling

#### Oleic acid % (18:1) and linolenic acid % (18:3)

Oleic and linolenic acid are important bioactive compounds that contribute to the oil composition of *B*. *napus*, whereas linolenic acid is an omega-3 fatty acid that is important for animal nutrition. The maximum oleic acid recorded for Faisal canola was 68.4% at T9 Zn NPs 25 mg/L with seeds that had been treated with a foliar spray. The maximum for the canola variety Shiralee was 66.1% at T4 5 mg/L with foliar spray treatment. Our result is consistent with the previous report that oleic acid was increased to 49.1% from 45.6% in peanut on exposure to TiO NPs [64]. Zn NPs at low concentration triggered the growth of both canola varieties, which shows that Zn NPs may have a role in the regulation of plant growth for Faisal canola and Shiralee. On the other hand, the maximum linolenic acid recorded for Faisal canola was 12.6% at T7 25 mg/L with treated seeds with Zn NPs, while Shiralee showed the highest linolenic acid content (9.5%) in control plants Table 2. Increased linolenic acid in peanut crop was reported for exposure to 500mg/kg of Ag NPs reported by Rui et al. [65]. However, decrease of oleic acid was observed on such exposure to Ag NPs. Foliar salicylic acid and jasmonic acid applications improved the quality of soybean seed oil by reducing oleic acid and increasing linoleic acid and linolenic acid content [66]. Such exogenous utilization of SA significantly triggered oil production and improve quality under saline and non-saline conditions. Thus, investigation of the effects of Zn NPs on alteration of the compositions and contents of fatty acids in rapeseed (*B*. *napus*) seeds is of great importance. Very less literature is available on the effect of NPs on plant fatty acid profiling. In the last decade, engineered NPs had received considerable attention and become a promising candidate for improving crops yield. The effect of NPs varies from plant to plant and depends on size, shape, and concentration [23].

#### Erucic acid % (C22:1) and glucosinolate content

Near-Infrared Reflectance Spectroscopy (NIRS) offers a hasty, non-destructive, and immediate investigation of fatty acid profiling, erucic acid and glucosinolate content. Different researchers have reported the convenient practice of NIRS for the investigation of different fatty acid profiles of *B*. species [67]. We applied this technique and the highest erucic acid recorded in Faisal canola was 1.7% at T2 Zn NPs 5 mg/L treated seed, while the same treatment exhibited an inhibitory effect on erucic acid in Faisal canola. On the other hand, the canola variety Shiralee showed consistent and significant increases in erucic acid on exposure to Zn NPs. Glucosinolate is a sulphur-comprising compound and an undesirable factor in canola oil. The results showed that exposure to Zn NPs led to a significant decrease in glucosinolate in *B*. *napus* variety Faisal Canola in contrast with the glucosinolate increase seen in Shiralee Table 2. Taken together with the results of content of fatty acid upon exposure to Zn NPs 5 mg/L, 15 mg/L and 25 mg/L suggested that the presence of Zn NPs reduce the erucic acid and glucosinolate content in rapeseed (*B*. *napus*) varieties and might cause a potential impact on human health. To our knowledge, this is the first study to investigate the effect of metallic nanoparticles on fatty acid profiling of *B*. *napus*.

## Conclusion

Green synthesis is one of the cost-effective and naturally safe way to get non-hazardous metallic NPs. Numbers of researchers, greenhouses, and field studies reported that germination and growth parameters of plants have been increased on exposure to nanoparticles. In the current study, fresh *M*. *arvensis* leaves were utilized for the synthesis of NPs, and the effects of the synthesized NPs were evaluated on *B*. *napus* varieties (Faisal canola and Shiralee) respectively. The results of this work confirmed that biochemical profiling was positively affected by the application of Zn NPs. The antioxidant enzymes showed a significant increase at all concentrations of Zn NPs, while nutritional analysis showed a notable change in its quantity, showing positive response towards Zn NPs. Thus, it has been concluded that Zn NPs could be used as a potential tool to increase important secondary metabolites production in medicinally important plants and improve the elemental/nutritional quality of oil crops. The fatty acid profile of *B*. *napus* showed a significant change in its profile under Zn NPs exposure. This data show that NPs positively affect plants when applied at appropriate concentrations such as 5 mg/L, 15 mg/L 25 mg/L. Plant physiological responses affect the interaction with nanoparticles, the results found in one crop might not be necessarily precise for other crops, which makes it necessary to study different types of plants. Therefore, the results of these studies encourage researchers to understand nanoparticle-plant surface interactions and the usefulness of green nanoparticles in plant systems.

## Supporting information

S1 Graphical abstract (DOCX)
