# Supplementary figures and images for: Nano zinc elicited biochemical characterization, nutritional assessment, antioxidant enzymes and fatty acid profiling of rapeseed
Source: PLoS One. 2020 Nov 10;15(11):e0241568. doi: 10.1371/journal.pone.0241568 (PMC7654759; doi:10.1371/journal.pone.0241568)

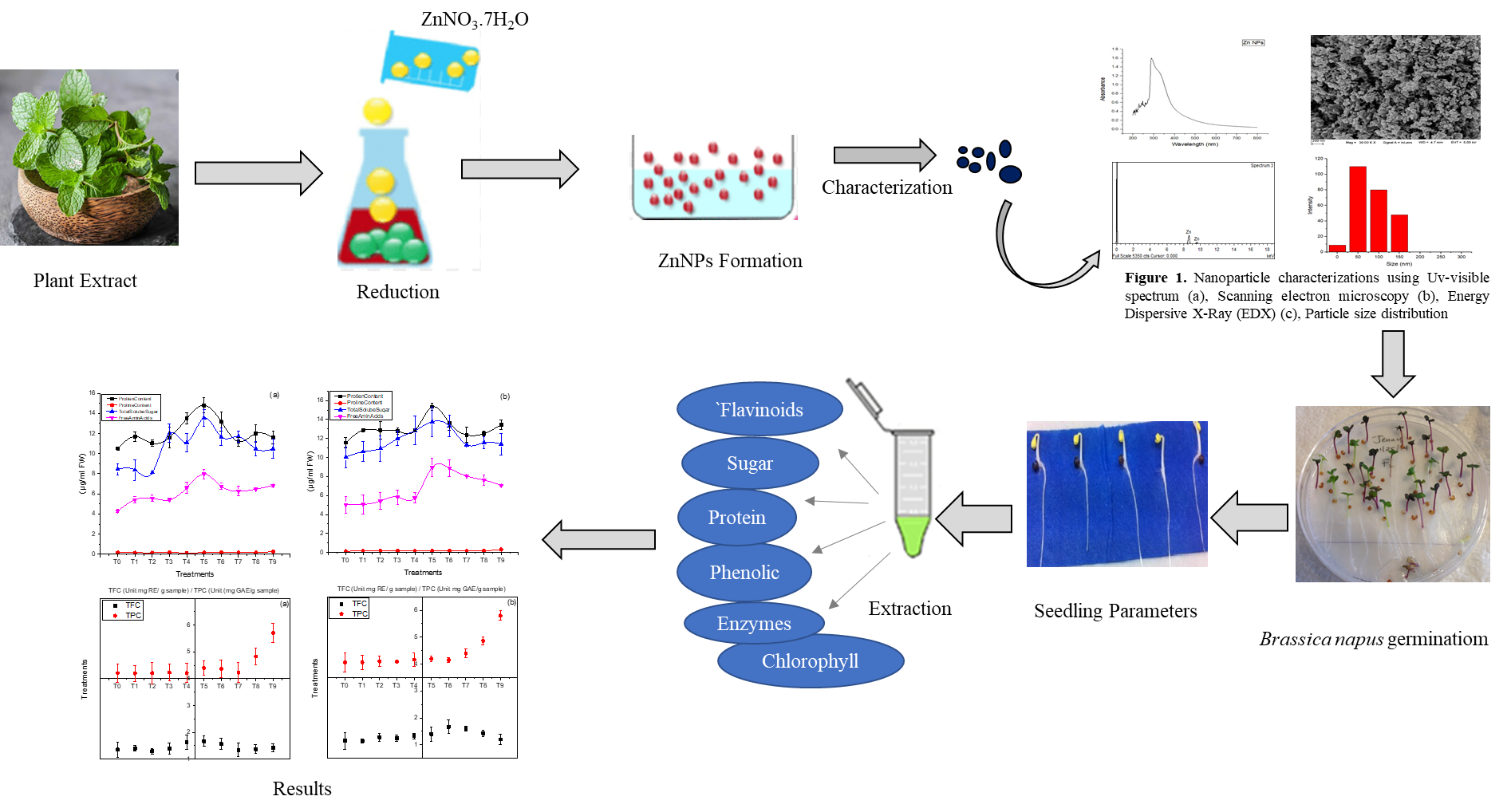

Supplement: S1 Graphical abstract — (DOCX) [file pone.0241568.s001.docx]
